# Identifying genomic targets for protein over-expression by “omics” analysis of Quiescent *Escherichia coli* cultures

**DOI:** 10.1186/s12934-017-0744-3

**Published:** 2017-07-28

**Authors:** Shubhashree Mahalik, Ashish Kumar Sharma, Priyanka Jain, Krishna Jyoti Mukherjee

**Affiliations:** School of Biotechnology, Jawaharlal Nehru University, New Delhi, 110067 India

**Keywords:** *E. coli*, Quiescence, Cellular stress response, *fis*, Transcriptomic profiling, Asparaginase expression

## Abstract

**Background:**

A cellular stress response is triggered upon induction of recombinant protein expression which feedback inhibits both growth as well as protein synthesis. In order to separate these two effects, it was decided to study “quiescent cultures” which continue to be metabolically active and express recombinant proteins even after growth cessation. The idea was to identify and up-regulate genes which are responsible for protein synthesis in the absence of growth. This would ensure that, even if growth were adversely affected post induction, there would be no attendant reduction in the protein expression capability of the cells. This strategy allowed us to design host strains, which did not grow better post induction but had significantly higher levels of protein expression.

**Results:**

A quiescent *Escherichia coli* culture, which is able to sustain recombinant protein expression in the absence of growth, was analyzed by transcriptomic and proteomic profiling. Many genes involved in carbon utilization, biosynthesis of building blocks and stress protection were found to be up-regulated in the quiescent phase. Analysis of the global regulators showed that *fis*, which tends to get down-regulated as the cells enter stationary phase, remained up-regulated throughout the non-growing quiescent phase. The downstream genes regulated by *fis* like *carB*, *fadB*, *nrfA*, *narH* and *queA* were also up-regulated in the quiescent phase which could be the reason behind the higher metabolic activity and protein expression ability of these non-growing cells. To test this hypothesis, we co-expressed *fis* in a control culture expressing recombinant l-asparaginase and observed a significantly higher buildup of l-asparaginase in the culture medium.

**Conclusions:**

This work represents an important breakthrough in the design of a superior host platform where a gene not directly associated with protein synthesis was used to generate a phenotype having higher protein expression capability. Many alternative gene targets were also identified which may have beneficial effects on expression ability.

**Electronic supplementary material:**

The online version of this article (doi:10.1186/s12934-017-0744-3) contains supplementary material, which is available to authorized users.

## Background

A key area, which has seen enormous growth in the past two decades, is the design of improved systems for expressing recombinant proteins. The availability of several expression hosts, strong titrable and inducible promoters to reduce inclusion body formation, fusion tags for efficient purification and solubilization, signal peptides for desired localization, and finally the high levels of productivity all ensure that the bacterial system remains the most extensively used system for this purpose [1–4]. In spite of these developments, protein expression remains a challenge with poor product yields often being a major impediment to successful commercialization of many recombinant products.

Recombinant protein over-expression elicits a stress response which down-regulates genes for substrate uptake, rRNA synthesis, energy metabolism and many others, all of which are critical for both growth and protein production [5]. It is therefore, common to observe the cessation of both, growth as well as recombinant protein synthesis, within a few hours post induction, which is the primary reason behind the poor yields, obtained in recombinant cultures [6]. Since protein synthesis and growth are inextricably linked processes, the usual strategy to extend the production phase has been to attempt to recover growth and hence protein expression by up-regulating these genes by knock-ins or plasmid based co-expression [7, 8]. However, if we were to focus only on those genes that feedback inhibits protein expression instead of alleviating the overall stress response, we could allow this response to selectively block growth without impeding protein production. This would have the dual advantage of diverting the metabolic flux towards product formation rather than biomass and also reducing the substrate and energy requirements of the culture. Clearly, non-growing or slow growing cells would also have significantly lower oxygen demand and heat production rates, factors which are critical during high cell density fermentations [9]. Identifying such genes to design such a cellular platform is however extremely difficult in practice. Unlike metabolites, which are the products of single pathways, recombinant protein synthesis involves the use of multiple features of the cellular machinery. Transcription, translation, energy, amino acids, tRNAs etc. are all inputs to this process and these are intricately linked to the process of growth. Also the control network of *E. coli* is a complex hierarchy, which is not fully elucidated [10, 11]. Separating genes controlling protein synthesis from those affecting growth is thus problematic.

One way out would be the availability of a quiescent culture where protein synthesis is uncoupled from growth, this could then be analyzed for its transcriptome and proteome profile with respect to a control culture in order to provide useful leads. A first step in the design of such a quiescent cell expression system was the identification of Rcd, a small RNA whose over-expression led to growth cessation and simultaneous increase in recombinant ScFv expression [12]. Further studies showed that Rcd acts by binding with tryptophanase and increases its affinity for tryptophan, leading to higher rates of indole formation [13]. We, therefore, developed a simpler chemical method of inducing quiescence by a controlled addition of indole into the bioreactor that led to successful protein expression in the quiescent phase [14]. Once this system was available we performed a comparative transcriptomic analysis of this indole induced quiescent cultures with non-quiescent control cultures to identify the differentially up and down-regulated genes. This was followed by looking at the proteomic profiles, since non-dividing quiescent cells may continue to harbor proteins long after their synthesis has stopped. These studies revealed the differential changes in the mRNA expression levels of transcriptional regulators, which are the nodes that control cellular metabolism, growth and protein expression. These genes were then utilized for the design of an improved platform for recombinant l-asparaginase (l-Asp) expression.

## Results

### Expression of l-Asp from quiescent *E. coli* W3110hnsΔ93-1 strain

**Fig. 1:**
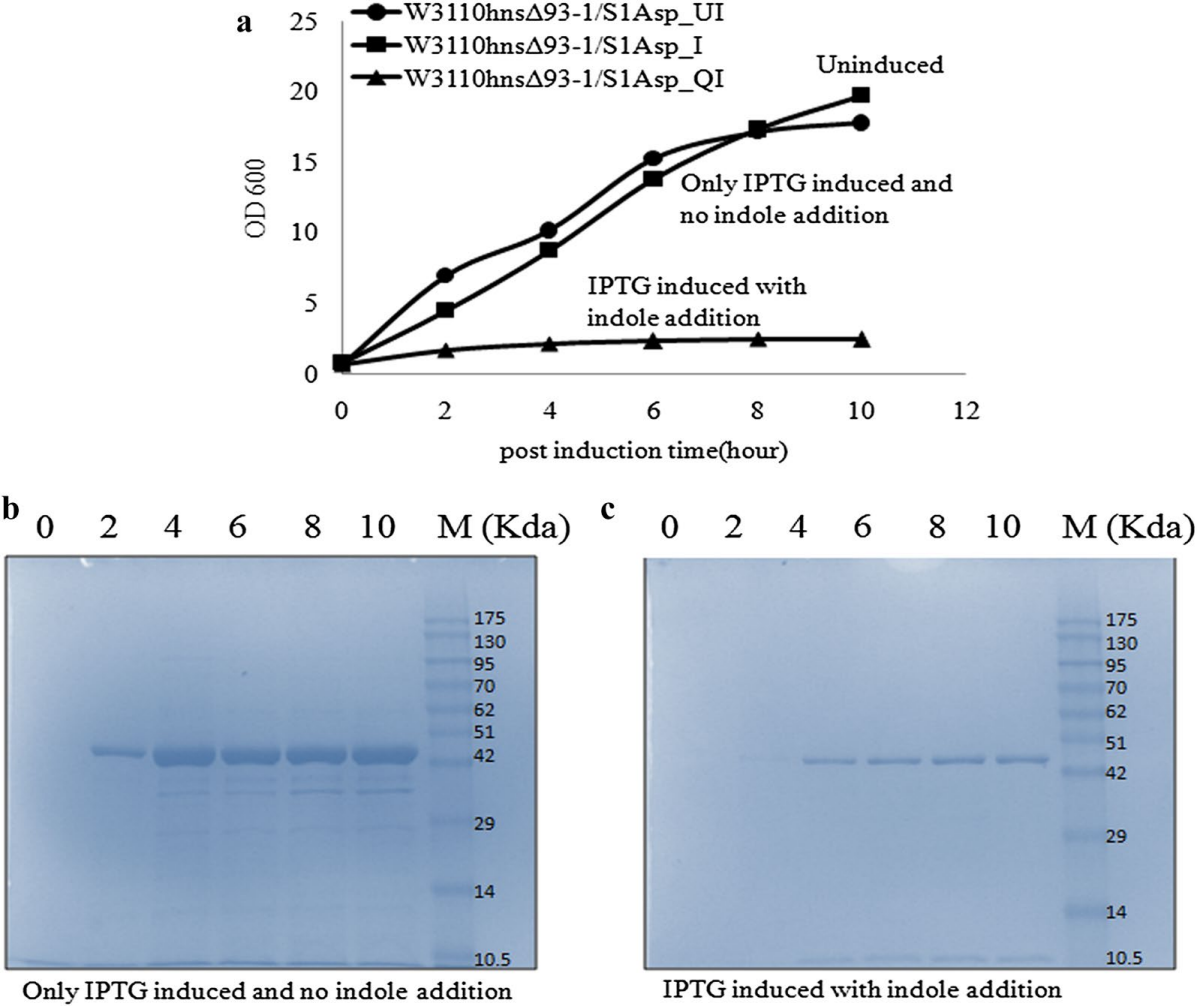
**a** Growth profile of W3110hnsΔ93-1 with and without indole treatment; SDS PAGE showing Post induction (0–10 h) extracellular expression profile of l-Asp **b** without indole **c** with indole addition. UI-uninduced, I-IPTG induction (without indole), QI-Indole + IPTG addition (with indole addition). The values of protein molecular weight markers are represented in kDa

After optimizing the procedure for indole addition, we analyzed the expression of l-Asp in indole treated cultures. For these experiments, 3.5 mM indole was used to induce quiescence in *E. coli* W3110hnsΔ93-1 cells [14], Three shake flask cultures were set up; an uninduced control culture without indole or IPTG (W3110hnsΔ93-1_UI), a second induced control culture with IPTG but no indole (W3110hnsΔ93-1_I) and a third test culture treated with indole for quiescence and then induced with IPTG for l-Asp expression (W3110hnsΔ93-1_QI). Very little difference was observed in the growth profile of the two control cultures (induced and uninduced; both without indole), in comparison to quiescent culture expressing l-Asp, which showed a significant decline in biomass formation. These experiments were repeated multiple times and a typical OD_600_ profile is shown in Fig. 1a. We compared the ability of W3110hnsΔ93-1 cells to express extracellular l-Asp under normal and quiescent condition and observed that the normal cells (W3110hnsΔ93-1_I) showed better expression as compared to quiescent cells. This can be attributed primarily to the significantly lower biomass concentrations obtained in quiescent cultures. However, the slow buildup of l-Asp in the supernatant of the quiescent cells did demonstrate the ‘proof of principle’ that quiescent cells do have the ability to express recombinant proteins under non-growing conditions (Fig. 1b, c).

### High cell density cultivation of quiescent culture

The next step was to induce quiescence by indole addition at high cell density where we could observe its stability and sustained expression capability. Several reactor runs were undertaken to estimate the metabolic activity and expression potential during quiescence. For this we ensured that the cellular environment was conducive to product formation by controlling the pH, dissolved oxygen (DO) and feeding rate. The activity of l-Asp was checked by a standard colorimetric assay using Nessler’s Reagent [15]. This allowed us to compare the rate of expression of l-Asp in quiescent cultures with control.

**Fig. 2:**
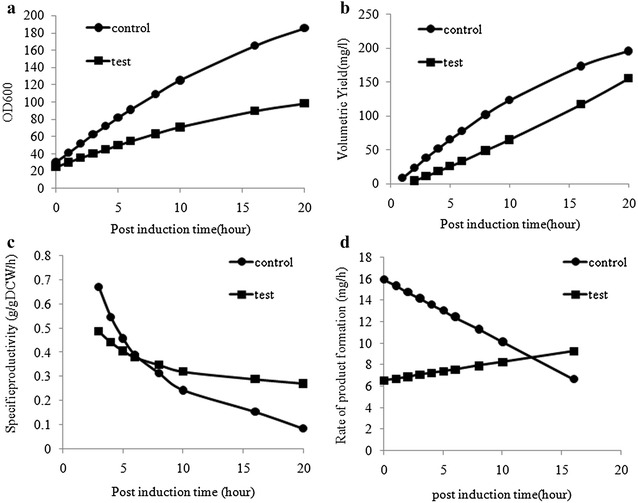
**a** Growth profile of Control (W3110hnsΔ93-1 without indole addition, C) and Quiescent (W3110hnsΔ93-1 treated with Indole, T) cells; Plot showing (**b**) Volumetric yield (**c**) Specific productivity (q_p_) (**d**) rate of product formation in control (W3110hnsΔ93-1 without indole addition, C) and Quiescent (W3110hnsΔ93-1 treated with Indole, T) cultures

The final OD_600_ of the control culture was 185, which was almost double the final biomass in quiescent culture (Fig. 2a). While the product concentrations in the supernatant were slightly higher in the control cultures (Fig. 2b), the specific product formation (q_p_) rates were better in the quiescent cells (Fig. 2c). Also the rate of product formation (dP/dt) in the control culture declined with time, whereas the quiescence culture could sustain the product formation rate till 20 h post induction (Fig. 2d). Thus, this strategy was able to achieve a state of quiescence where growth and product formation kinetics were decoupled and metabolic fluxes were diverted towards recombinant protein formation. To locate the targets that are specifically responsible for allowing protein expression to continue unhindered during the quiescent phase, it was decided to do transcriptomic and proteomic profiling and identify the differentially expressed genes and proteins. The idea was to try and separate the genes involved in growth cessation from those which impact specifically on protein expression.

### Transcriptomic profiling to identify genes responsible for diverting the metabolic flux specifically towards product formation

Earlier studies on transcriptomic profiling in our lab have analyzed the post induction response of cultures expressing recombinant proteins [6, 16]. Both specific growth rate (µ) and specific product formation rate (q_p_) decline simultaneously during the post induction phase of recombinant protein expression. So it is difficult to identify those genes that are specifically responsible for the drop in expression capacity but not growth. Quiescent cells thus, provide an ideal platform where such genes can be separated from those responsible for growth cessation. To locate the appropriate window to study, we selected a phase of declining µ post induction and compared the change in q_p_ in both control and quiescent cultures. We observed that when µ declined from 0.1 to 0.05 h^−1^ the q_p_ of the control culture declined sharply, whereas the q_p_ of the quiescent cells remained more or less constant at a much higher value (Table 1). Samples for transcriptomic analysis were therefore taken when the µ values were 0.1, 0.08 and 0.05 h^−1^.

**Table 1:** Post induction specific growth rate and the corresponding specific production rate of control (without indole) and Quiescent (indole treated) cultures

Specific growth rate (h^−1^)	q_p__control culture (g/gDCW/h)	q_p__test culture (g/gDCW/h)
0.1	0.456	0.404
0.08	0.241	0.318
0.05	0.082	0.269

l-Asparaginase expression is induced with IPTG and *fis* is induced with l-arabinose

### Impact of quiescence on cellular physiology

**Fig. 3:**
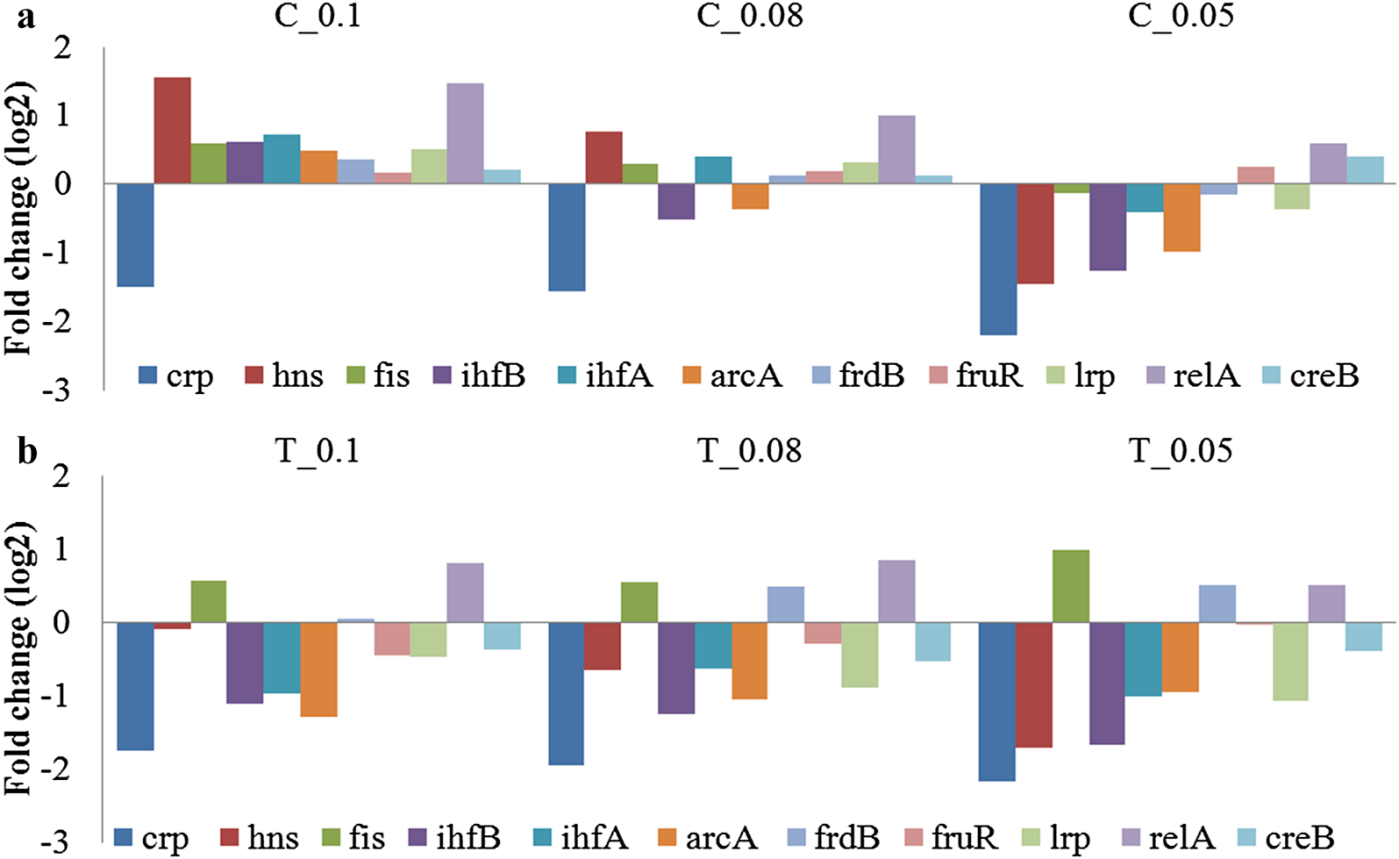
Transcriptomic profiles of Control and Quiescent cultures showing differentially expressed master regulators of cellular metabolism across declining specific growth rate. **a** Control (W3110hnsΔ93-1 without indole addition) **b** Test (Quiescent culture, W3110hnsΔ93-1 treated with Indole); 0.1, 0.08, 0.05 represents the post induction specific growth rates

An important aspect of the quiescent cell physiology is their sustained protein expression capability compared to control. To get a holistic picture and identify the genes responsible, we looked at the difference in the transcriptomic profile of the control and quiescent cultures with respect to the genes coding for regulatory proteins (Fig. 3). The difference in transcriptomic profile was determined on the basis of fold change in mRNA level, which refers to the change in gene expression levels between pre and post induction samples due to the stress associated with recombinant protein expression. Control cultures have a typical stress response and hence a characteristic fold change pattern whereas the quiescent cultures (Test) have an altered stress response and hence a different fold change pattern. It is this difference that allowed us to identify the differentially expressed genes. Even though the experiments were carried out with a W3110hnsΔ93-1 strain, which produces a truncated H-NS protein, the expression pattern of the H-NS gene was analyzed, since the H-NS mutation may lead to the differential expression of several proteins.

More important were the genes expressing regulatory proteins, as their differential expression was critical to the generation of quiescence. It was observed that in the control culture, most of these regulatory genes were up-regulated in the early phase of induction, when the specific growth rate was high. With declining specific growth rates, the expression of most of these genes got down-regulated. In contrast, in the quiescent culture most of these regulators were down-regulated from the start and the pattern did not change significantly with time. However, three genes i.e. *fis*, *frdB* and *relA* remained up-regulated in the quiescent cells and of these *fis* showed the maximum deviation from control profile. We therefore, focused on *fis* whose mRNA and protein levels are known to peak during early logarithmic phase, and decrease soon thereafter, becoming nearly undetectable as cells enter the stationary phase, an expression pattern referred to as growth phase-dependent regulation [17]. As expected this pattern was observed in the transcriptomic profile of the control culture. However, we obtained an interesting profile for quiescent culture where, irrespective of the slowdown in specific growth rate, there was an elevated level of *fis* throughout the period of quiescence. Since recombinant protein expression levels also remained high, we can hypothesize that *fis* up-regulation is responsible for this phenotype. It possibly counteracts the inhibitory effects of ppGpp, which is responsible for down-regulating rRNA synthesis during slow growing conditions, and thus, helps in enhancing recombinant protein expression in the quiescent phase.

**Fig. 4:**
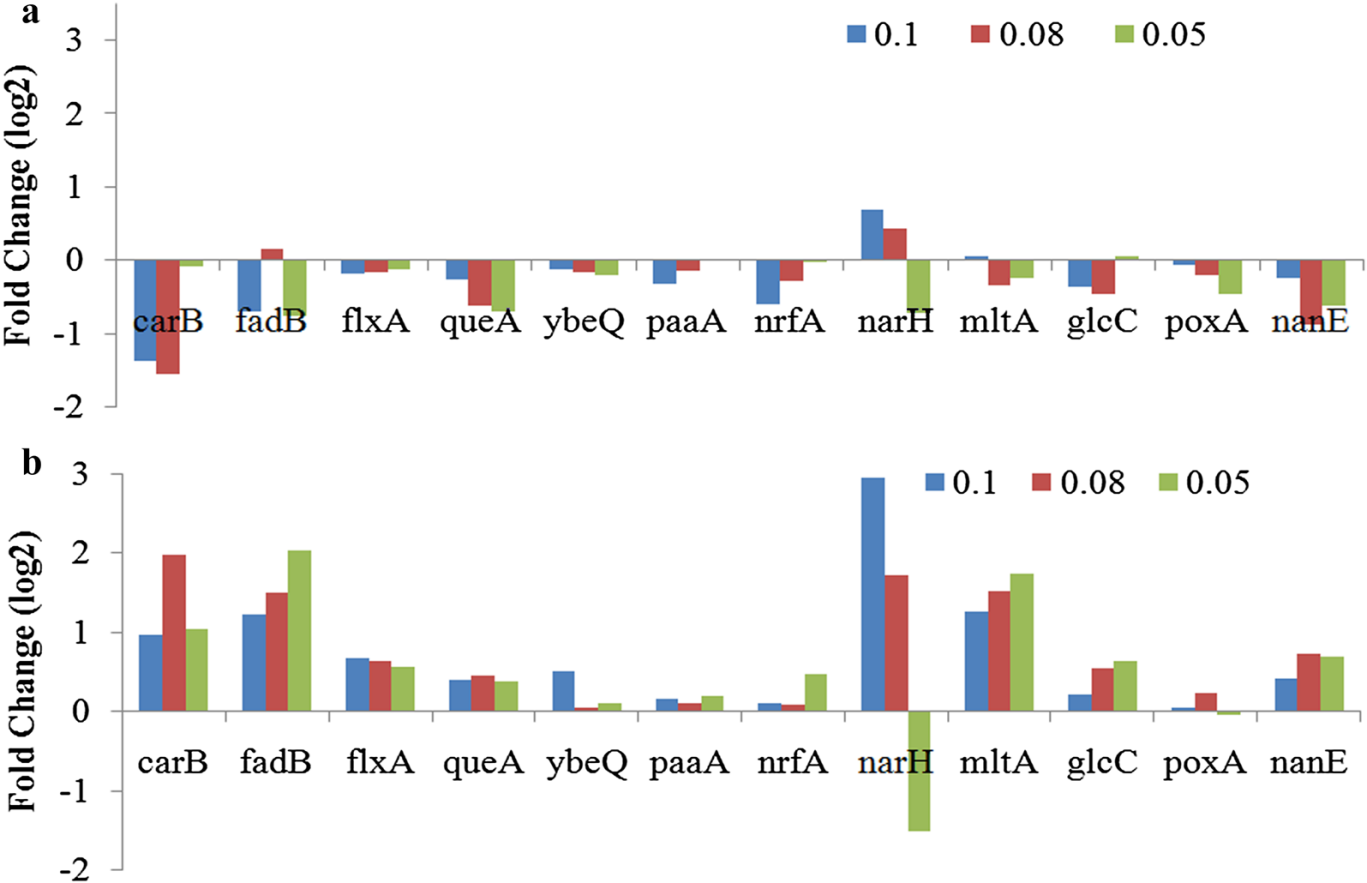
Differential expression of genes under directs regulation of *fis*. **a** Control (W3110hnsΔ93-1 without indole addition) **b** Test (Quiescent culture, W3110hnsΔ93-1 treated with Indole); 0.1, 0.08, 0.05 represents the post induction specific growth rates

To prove the hypothesis that *fis* plays a critical role in maintaining cellular metabolic activity; we analyzed those genes that are under direct regulation of *fis*. The list of genes under direct regulation of *fis* was obtained from the Ecocyc database and is also available in literature [18]. Figure 4 shows that these genes were also down-regulated in the control, compared to the quiescent cells. Thus, *carB* (amino acid biosynthesis and de novo UMP biosynthesis), *fadB* (beta-oxidation of fatty acid for generation of energy equivalents), *nrfA* and *narH* (electron transport chain) were all significantly up regulated in the quiescent culture compared to the control. This up regulation indicates that the quiescent cells were metabolically active and were able to perform all the critical functions required for maintaining cellular health and expression capacity even during the non-dividing stage.

### Proteomic profile of the quiescent culture

Earlier reports have shown that the proteome can differ significantly from the transcriptome due to variations in translational efficiencies and stability of the proteins [19]. In quiescent cells, where in the absence of cell division the protein concentrations do not get diluted out, stable proteins can remain at high levels long after the transcriptional machinery has shut down. Thus, the proteomic profiles of quiescent cells can be significantly different from the transcriptome. Samples from fed batch fermentations, induced for l-Asp expression, with and without indole addition, were collected at three time points corresponding to specific growth rates of 0.1, 0.08 and 0.05 h^−1^ post induction, thus, matching the transcriptomic profile time points. The sample from fed batch fermentation where indole was not added was taken as control (C) and those with indole addition were taken as test samples (T). Pre induction samples were taken as a control for every run. 2D PAGE and subsequent protein identification was carried out as described in materials and methods. The PANTHER Classification system was used to biologically classify the identified proteins on the basis of their Molecular Functions, role in Biological Process, Cellular Location and Pathway to which they belong. In the control culture, 278 distinct spots were obtained, while 259 spots were seen in the quiescent culture. Using databases, we could identify 116 proteins in the control set while only 84 proteins could be identified in the quiescent culture.

**Fig. 5:**
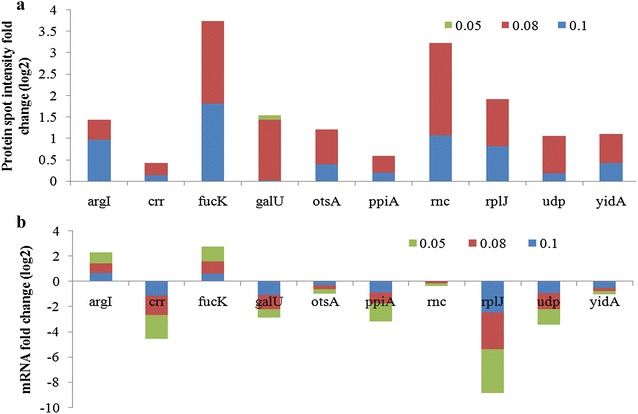
Two-way analysis of transcriptome and proteome of Control and Quiescent culture. The subset of protein represented here are present in differential amounts in Quiescent cultures (W3110hnsΔ93-1 with indole addition) but absent in the Control (W3110hnsΔ93-1 without indole addition. **a** Protein spot intensity in Quiescent culture, whereas **b** corresponding mRNA Transcript level of protein spots; 0.1, 0.08, 0.05 represents the post induction specific growth rates

The protein spots that were considered for this study were those specifically present in the quiescent culture but either absent or present in extremely low concentrations in the control culture (Fig. 5). Indole not only induces quiescence but also acts as a signalling molecule that has a widespread effect on cellular physiology. This part was critical since the proteomic profile would identify targets that have been specifically hit by indole. Indole toxicity would generate a metabolic response but simultaneously it would reduce the stress associated with recombinant protein production by slowing the growth rate. So there was a need to differentiate between the two effects viz indole toxicity and generation of quiescent phenotype with a concomitant reduction in the cellular stress response. This would allow us to choose the best gene targets to alleviate the deleterious effects of the cellular stress response on recombinant protein production without the attendant ill effects of indole toxicity. A 2-way comparison between the transcriptomic and proteomic profiles of both control and quiescent conditions for this set would confirm their role in quiescence and enhanced expression capability. In quiescent cultures, since cells are in a non growing stage, the energy requirements are low, but the glycolytic pathway remains active and up-regulation of Crr, FucK, GalU (carbon utilization) indicates that quiescent cells have active pathways for utilization of alternative carbon sources to fuel the glycolytic pathway. Similarly Udp protein was found to be up-regulated which is known to be involved in the pathway that utilizes nucleosides as a carbon and energy source. This is again an indication that the quiescent cell is using alternative sources of energy in the absence of an active TCA cycle. ArgI, (biosynthesis of building blocks), OtsA (stress protection), TatB (protein transporter) proteins were observed only in the proteome of the quiescent cells which explains how, even in the non dividing stage, quiescent cells retain a high basal level of metabolic activity and several proteins of critical pathways are up-regulated. Also, unlike the control culture, proteins related to recombinant protein expression, folding and processing like RplJ, Rnc, PpiA were present in high amounts, which could be the reason for quiescent cells having an enhanced protein production rate. The proteomic profiling of quiescent cells thus helped us to identify the set of proteins whose presence allowed it to retain its metabolic activity along with its expression ability, even in the absence of growth. While the transcriptomic analysis did identify some genes whose up-regulation may be responsible for this desired phenotype, the proteomic data constituted a direct proof. Thus proteomic analysis helped in identifying proteins that did not decline, due to reasons of protein stability; even if mRNA levels got down-regulated. Such proteins by virtue of their presence in fairly high concentrations could have a critical role in generating the quiescent phenotype.

The differential proteome analysis using 2-DE suggested that several key changes take place in cellular physiology including carbon and energy metabolism, building block biosynthesis, protein translation, folding, and cell protection. Proteome analysis not only validated the transcriptomic data but also offered better insights for further genetic modifications that may be required for designing a host with enhanced protein expression capability.

### Effects of *fis* co-expression on recombinant protein expression

A fairly large number of leads were obtained from the differential transcriptomic and proteomic profile of the quiescent cells with respect to control. Among these *fis* was chosen as the most promising candidate for up-regulation, given its role as a global regulator and multiple effects on cellular physiology. Thus, an important function of *fis* is the activation of stable RNA (rRNA and tRNA) and induction of related promoters on nutritional shift-up [20, 21], which would increase the capacity of the translational machinery. Also *fis* enhances energy metabolism by growth phase stimulation of the *nuo* and *ndh* operons to yield high ATP and maintain redox balance [22]. Since recombinant protein production is an energy intensive process and down-regulation of ribosome biosynthesis and translational machinery is known to be one of the major bottlenecks in recombinant protein synthesis [23], it was decided to co-express *fis* along with a recombinant protein (l-Asp) to test its effect on protein expression.

**Fig. 6:**
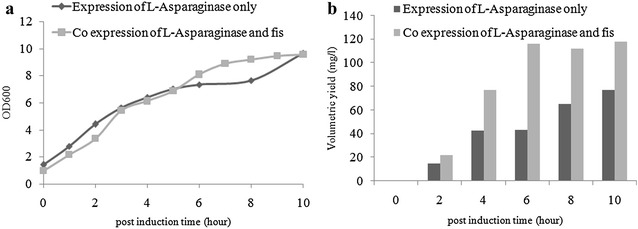
**a** Growth profile, **b** Production profile of W3110 cells co-expressing l-Asp with *fis*

Cell growth was monitored and no significant difference was observed in the growth profile of control and *fis* co-expressed cultures (Fig. 6a). Protein quantification of supernatant samples was performed and we observed that *fis* over-expression led to drastic increase in l-Asp productivity (Fig. 6b). Clearly, *fis* which is involved in ribosome synthesis during rapid cell growth [24] and energy metabolism [22] has an important role in sustaining recombinant protein production.

## Discussion

Previous studies on transcriptomic profiling of high cell density cultures expressing recombinant proteins, have shown that a large number of genes are highly down-regulated post induction as a part of the cellular stress response [25, 26] which is the probable reason for decline in both growth and specific product formation rate [16]. We postulated that this stress response is a two-step process where, first, growth gets blocked and subsequently, this growth cessation leads to the down-regulation of genes involved in energy metabolism and protein translation. Indeed, a careful analysis of post induction growth and protein production profiles for many proteins shows that product formation continues for a slightly longer period than growth. These two effects could, therefore, be separated and a nuanced strategy, which selectively allows protein expression to continue, can be adopted, leading to a design of a superior expression platform.

l-Asparaginase was chosen as a model protein for this study because it first builds up in the periplasm and then leaches out as an extracellular product. Growth typically stops by the time l-asparaginase has accumulated in the periplasm and, therefore, in control, we see only a short period of extracellular production. With *fis* co-expression, this process was sustained for a significantly longer time, clearly demonstrating the validity of this approach.

The proteome of the quiescent cells showed many additional targets for up-regulation. Many of these are involved in carbon utilization, biosynthesis, energy metabolism, etc., all of which are critical for protein production. Since these genes are not under the control of *fis* they may have synergistic effects on expression capability.

## Conclusions

Systems biology tools offer a platform to understand the cellular physiology and plan strategies to design better platforms for enhanced production of recombinant proteins. From the combined -omics analysis and co-expression studies, we could conclude that the cell strategically respond to stress associated to recombinant protein production by altering expression at mRNA and protein levels of key genes. The potential of such modified strains could thus be explored for the expression of toxic proteins whose induction leads to a sharp drop in growth, leading in turn to very low levels of expression.

## Methods

### Strains and plasmid

The strains and plasmids used in this study are listed in Additional file 1: Table S1. All the strains were cultured aerobically in commercially available Terrific Broth (TB) medium supplemented with 10 mM MgSO_4_ and 0.4% glycerol at 37 °C with constant shaking at 200 RPM. The concentrations of antibiotics used were ampicillin 100 µg/ml (1×) and kanamycin 50 µg/ml (1×).

### Construction of plasmids and recombinant DNA techniques

Recombinant DNA work was carried out according to standard protocols described in Maniatis et al. [27]. Qiagen plasmid isolation kit was used to isolate all plasmids. Restriction endonucleases were purchased from Fermentas (Waltham, MA, USA) and digestions were performed accordingly. Qiagen gel extraction kit was used to purify digested vectors and PCR fragments from agarose gels. DNA ligations and subsequent transformations into competent *E. coli* DH5α were carried out according to standard protocols. The model protein used for this work was l-asparaginase (l-Asp). The detailed construction of the recombinant l-Asp plasmid pMALS1Asp used in this study has been described in Additional file 1: Figure S1.

### Fed batch of quiescent cells

For fed batch cultivation, freshly transformed W3110hnsΔ93-1 carrying the recombinant pMALS1Asp plasmid was inoculated in 10 ml of TB medium containing 100 µg/ml ampicillin and grown overnight. This culture was used to inoculate 200 ml TB medium having the same antibiotic concentration and grown for 8–10 h till an OD_600_ of 6–7. This was used as an inoculum for the fermenter containing 2 l TB medium. The initial media composition for the batch was TB medium supplemented with 0.4% glycerol and 10 mM MgSO_4_. The temperature, pH and DO were set at 37 °C, 7.0 and 40%, respectively. The initial stirrer speed, airflow, pH control and automatic DO control was the same for all the runs. Antifoam was used occasionally as required. The feed composition was 18% glycerol, 12% yeast extract, 12% tryptone and 10 mM MgSO_4_.

Addition of indole is a critical step to induce quiescence. Several parameters had to be monitored before and during indole addition. Among them pH and RPM were critical. Once the batch culture OD_600_ reached 8–10 the feed was started at a constant flow rate of 46 ml/h. The increase in OD_600_ was monitored every 30 min and the specific growth rate (µ) was calculated. The culture was induced using 1 mM IPTG when the µ value fell to 0.25 h^−1^. Simultaneously the indole pump was started at 0.16 ml/min and run for ~2 h. Any decline in oxygen transfer rate (OTR) as indicated by a rise in DO and a subsequent fall in RPM along with decline in pH indicated the onset of indole toxicity and therefore, the indole feed was stopped. The pH, RPM and DO recovered within 30 min of stoppage. 1 mM IPTG and 8 ml/l indole was also added to the feed tank to prevent their dilution in the bioreactor. Samples were collected hourly for analysis. Similar conditions were used for the control culture where no indole was added.

### Microarray experiment

Samples from the fed batch fermentations of quiescent and control cultures were collected at three time points post induction corresponding to specific growth rates of 0.1, 0.08 and 0.05 h^−1^. The samples from cultures without indole addition are taken as control (C) and those with indole addition were taken as the test (T). Pre induction samples were taken as an additional control for every run. The cDNA synthesis, labelling (biotin) and hybridization (Affymetrix GeneChip *E. coli* genome 2.0 array) were performed according to the Affymetrix GeneChip expression analysis protocols. Washing, staining and amplification were carried out in an AffymetrixGeneChip^®^ Fluidics Station 450. AffymetrixGeneChip^®^ scanner 3000 was used to scan the microarrays. Quantification and acquisition of array images were done using Affymetrix Gene Chip Operating Software (GCOS) version 1.4. Three types of detection calls (i.e., present, absent, or marginal) were calculated using statistical expression algorithm and average normalization was performed. Hybridization and spike controls were used.

Subsequent data analysis was performed using GeneSpring GX11.5 software (Agilent Technologies, USA). RMA algorithm was used for data summarization [28] and quality control of samples was assessed by principle component analysis (PCA). Fold change was calculated with respect to the uninduced control (0 h). Normalized signal intensities of each gene on chips were converted to log_2_ values, and compared between experiments.

The microarray data has been deposited in the Gene Expression Omnibus database at NCBI (GEO: www.ncbi.nlm.nih.gov/geo/query/acc.cgi?acc=GSE29486) under the Accession Number GSE29486.

### Proteomic profiling

Preparation of crude protein extracts was performed as per BioRad’s recommended protocol with minor modification. Briefly, cells were collected, washed, and resuspended in Tris-Sucrose buffer. The suspension was then mixed with 200 μl of lysis solution (7 M urea, 2 M thiourea, 4% CHAPS; freshly prepared by supplementation with 10 mg/ml dithiothreitol (DTT) and 10 μl/ml protease inhibitor PMSF). Cells were disrupted on ice by sonic disintegration using Hielscher UP200S sonicator equipped with a micro tip. Collection of whole cell lysates was performed by centrifugation at 15,000 RPM for 30 min at 4 °C. The lysate was TCA precipitated and the precipitated protein was resuspended in Rehydration buffer (8 M urea, 4% CHAPS, 0.001% bromophenol blue and 65 mM DTT) containing 1% 3–10 IPG ampholyte buffer. Bradford’s method was used for quantification of protein amounts using bovine serum albumin as a standard. Two-dimensional gel electrophoresis was carried out using 2-D Electrophoresis System (BioRad, USA). 400 μg of bacterial protein extract was mixed with 400 μl of rehydration buffer. The rehydrated sample was loaded on to 18-cm IPG strips with pH range of 3-10. Isoelectric focusing was done on BioRad Protean IEF Cell using recommended protocol. After the complete process was accomplished, the equilibrated strip was subjected to the second dimensional separation (Protean II Xi Cell) using a SDS-PAGE (12%). The gels were stained with either Coomassie Brilliant Blue R-250 or Sypro Ruby Red according to the standard recommendation. After staining, gel images were acquired using Molecular Imager PharosFX™ System (BioRad). Differential analysis including spot detection, normalization, quantification, and matching was performed by PDQuest Advanced version 8.1.1 (BioRad) software tool. Mass spectrometry and peptide mass fingerprinting (PMF) analysis were carried out by ReflexIV, BrukerDaltonics, Germany MALDI-TOF/TOF Analyser. The BioTool 2.0 software (BrukerDaltonics) integrated with the MASCOT 2.2 search engine (MatrixScience, http://www.matrixscience.com/) was used for spot proteins identification by querying the trypsin-digested peptide fragment data using the reference database NCBInr 20121103 (21332039 sequences; 7307542895 residues). The taxonomy selected was *Escherichia coli* (383979 sequences). The search parameters used were; type of search: peptide mass fingerprint, enzyme: trypsin, fixed modifications: carbamidomethyl (C), variable modifications: oxidation (M), mass values: monoisotopic protein mass: unrestricted, peptide mass tolerance: ±100 ppm, peptide charge state: 1+ , max missed cleavages: 1. Bruker Daltonics BioTools Version 2.2 software was used for interpretation of MS data.

### Cloning and co-expression of regulatory gene *fis*

*fis* Gene was chosen from transcriptomic profile analysis for co-expression studies. It was cloned in pPROLar.A122 which is part of the PRO Bacterial Expression System. The plasmid is compatible for co-expression with pMALS1Asp. The details of the cloning procedure are provided in the Additional file 1: Figure S2.

*Escherichia coli* W3110 cells were co-transformed with pMALS1Asp and pPROfis and transformants were grown overnight in 10 ml TB medium with 50 µg/ml of ampicillin (0.5×) and 25 µg/ml of kanamycin (0.5×) at 37 °C with shaking at 200 RPM. 500 µl of primary culture was used to inoculate four 500 ml flasks each containing 50 ml TB medium so that the experiments could be run in duplicates. The recombinant cultures were induced either with 1 mM IPTG for l-Asp expression (control) or both 1 mM IPTG and 0.2% l-arabinose to co-express both genes (test). In these experiments no indole was added to induce quiescence. Post induction samples were collected every 2 h. Cell growth was monitored by measuring OD at 600 nm. Samples were analyzed by SDS PAGE and protein quantification was performed by densitometric scanning of Sypro Ruby stained gels.

## Additional file

**Additional file 1.** Additional figures and tables.
