# Supplementary material for: Identifying genomic targets for protein over-expression by “omics” analysis of Quiescent Escherichia coli cultures
Source: Microb Cell Fact. 2017 Jul 28;16:133. doi: 10.1186/s12934-017-0744-3 (PMC5534100; doi:10.1186/s12934-017-0744-3)
Supplement: Supplementary file 1 — Additional file 1. Additional figures and tables. [file 12934_2017_744_MOESM1_ESM.docx]

**Identifying genomic targets for protein over-expression by “omics” analysis of Quiescent *Escherichia coli* cultures**

**Shubhashree Mahalik, Ashish Kumar Sharma, Priyanka Jain, Krishna Jyoti Mukherjee^*^**

*** Correspondence: Krishna Jyoti Mukherjee**: [kjmukherjee@mail.jnu.ac.in](mailto:kjmukherjee@mail.jnu.ac.in)

## Additional file 1: Figure S1

*ansB*^-^ was cloned under a comparatively weaker promoter in a low copy number plasmid so that sustained protein expression under quiescent conditions could be clearly observed. For this *ansB^-^* along with the *pelB* leader sequence was sub cloned in the pMAL-p2X vector. The vector pPNHAsp(Khushoo et al., 2004) was double digested with *XbaI*/*HindIII* restriction enzymes resulting in a release of 1.153kb fallout corresponding to the rbs, *pelB* signal sequence and *ansB^-^* gene. Simultaneously the pMAL-p2X vector was linearized with *XbaI*/*HindIII* restriction enzymes. The purified 1.153kb segment was ligated into linearized pMAL-p2X vector. The positive clones were identified by the 1153bp fallout corresponding to the size of rbs + *pelB* signal sequence + *ansB*^-^ gene. Further confirmation was done by DNA sequencing. The confirmed clone was labeled as pMALS1Asp.


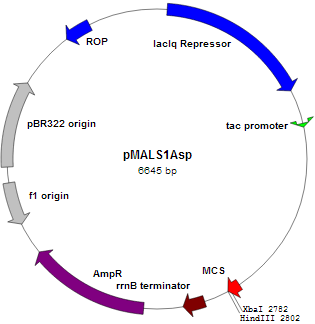


**Figure S1.** Construction of recombinant plasmid pMALS1Asp

## Additional file 1: Figure S2

The Factor of Inversion (*fis*) gene was amplified from the genome of *E.coli* W3110 by PCR using primer *fis*F and *fis*R. The PCR cycles produced an amplified fragment of 297bp which was digested with restriction enzymes *BamHI* and *XbaI*. The PCR product was purified and ligated to plasmid pPROLarA.122 also digested with the same restriction enzymes. The positive clones were selected by shift in plasmid size and further confirmed by restriction digestion leading to a fallout of 297bp. The insert was confirmed by sequencing and the plasmid labeled as pPROfis.

Primers Used in Cloning of fis:

| Name | Sequence (5’ 3’) |
| --- | --- |
| *fis*_F | CGGGATCCATGTTCGAACAACGCGTAAA |
| *fis*_R | GCTCTAGATTAGTTCATGCCGTATTTTT |

**Figure S2.** Construction of recombinant plasmid pPROfis

## Additional file 1: Table 1.

## Strains & Plasmids used in the study

| ***E.coli* strain** | **Genotype** | **Source** |
| --- | --- | --- |
| W3110hnsΔ93-1 | E.coli K12 F- λ- rph-1 hnsΔ93 INV(rrnD, rrnE) | Dr.David Summers, Cambridge, UK. |
| **Plasmids** | **Description** | **Source** |
| pMALS1Asp | L-Asparaginase (ansB) gene cloned under tac promoter in pMAL-p2X vector | This study |
| pPROfis | fis gene cloned under araBAD promoter in pPROLar.A vector | This study |

## Additional file 1: Table 2.

**Spot Intensities of Cluster of proteins identified from 2D gels of Test (Quiescent, W3110hnsΔ93-1 treated with Indole) culture that are present in differential amounts in, Quiescent cultures but absent in the Control (W3110hnsΔ93-1 without indole addition). Spots enlisted here correspond to Figure 5. 0 represents Uninduced culture and 0.1, 0.08, 0.05 represents the post induction specific growth rates.**

| SSP | Protein ID | Mr | pI | 0 | 0.1 | 0.08 | 0.05 |
| --- | --- | --- | --- | --- | --- | --- | --- |
| 2401 | ArgI | 40.54 | 4.81 | 14282.5 | 37489.7 | 22868.2 | - |
| 1102 | Crr | 18.51 | 4.73 | 9394.1 | 10768.9 | 12395.1 | - |
| 1501 | FucK | 55.09 | 4.69 | 7499 | 45891.6 | 51955.6 | - |
| 4302 | GalU | 32.33 | 5.11 | 16209.1 | 16461.6 | 67186.5 | 17859.3 |
| 6502 | OtsA | 53.07 | 6.29 | 5194.4 | 7667.5 | 11753.8 | - |
| 8101 | PpiA | 18.77 | 9.04 | 19659.9 | 23857 | 29175.4 | - |
| 6204 | Rnc | 26.73 | 6.41 | 7615.7 | 22179.8 | 66085.1 | - |
| 8102 | RplJ | 17.7 | 9.04 | 43102.9 | 96935.9 | 130839.9 | - |
| 4105 | TatB | 18.77 | 5.13 | 9982.1 | 3671.4 | 12395.1 | - |
| 6201 | Udp | 27.57 | 5.87 | 30797 | 36852.1 | 73756.3 | - |
| 4201 | YeaZ | 25.67 | 5.01 | 8754.6 | 10274.5 | 20913.1 | - |
